# Exercise in the Management of Metabolic-Associated Fatty Liver Disease (MAFLD) in Adults: A Position Statement from Exercise and Sport Science Australia

**DOI:** 10.1007/s40279-023-01918-w

**Published:** 2023-09-11

**Authors:** Shelley E. Keating, Angelo Sabag, Kate Hallsworth, Ingrid J. Hickman, Graeme A. Macdonald, Jonathan G. Stine, Jacob George, Nathan A. Johnson

**Affiliations:** 1 School of Human Movement and Nutrition Sciences, The University of Queensland, Room 534, Bd 26B, St Lucia, Brisbane, QLD 4067 Australia; 2 Faculty of Medicine and Health, Discipline of Exercise and Sport Science, University of Sydney, Sydney, NSW Australia; 3 Charles Perkins Centre, University of Sydney, Camperdown, NSW Australia; 4 NICM Health Research Institute, Western Sydney University, Westmead, NSW Australia; 5 NIHR Newcastle Biomedical Research Centre, Newcastle Upon Tyne Hospitals NHS Foundation Trust, Newcastle Upon Tyne, UK; 6 Liver Unit, Newcastle Upon Tyne Hospitals NHS Foundation Trust, Newcastle Upon Tyne, UK; 7 Translational and Clinical Research Institute, Faculty of Medical Sciences, Newcastle University, Newcastle Upon Tyne, UK; 8 Department of Nutrition and Dietetics, Princess Alexandra Hospital, Brisbane, QLD Australia; 9 Faculty of Medicine, PA-Southside Clinical Unit, The University of Queensland, Brisbane, QLD Australia; 10 Department of Gastroenterology and Hepatology, Princess Alexandra Hospital, Brisbane, QLD Australia; 11 Division of Gastroenterology and Hepatology, Department of Medicine, The Pennsylvania State University- Milton S. Hershey Medical Center, Hershey, PA USA; 12 Department of Public Health Sciences, The Pennsylvania State University- College of Medicine, Hershey, PA USA; 13 Liver Center, The Pennsylvania State University- Milton S. Hershey Medical Center, Hershey, PA USA; 14 Cancer Institute, The Pennsylvania State University- Milton S. Hershey Medical Center, Hershey, PA USA; 15 Storr Liver Centre, The Westmead Institute for Medical Research and Westmead Hospital, University of Sydney, Sydney, NSW Australia

## Abstract

Metabolic-associated fatty liver disease (MAFLD) is the most prevalent chronic liver disease worldwide, affecting 25% of people globally and up to 80% of people with obesity. MAFLD is characterised by fat accumulation in the liver (hepatic steatosis) with varying degrees of inflammation and fibrosis. MAFLD is strongly linked with cardiometabolic disease and lifestyle-related cancers, in addition to heightened liver-related morbidity and mortality. This position statement examines evidence for exercise in the management of MAFLD and describes the role of the exercise professional in the context of the multi-disciplinary care team. The purpose of these guidelines is to equip the exercise professional with a broad understanding of the pathophysiological underpinnings of MAFLD, how it is diagnosed and managed in clinical practice, and to provide evidence- and consensus-based recommendations for exercise therapy in MAFLD management. The majority of research evidence indicates that 150–240 min per week of at least moderate-intensity aerobic exercise can reduce hepatic steatosis by ~ 2–4% (absolute reduction), but as little as 135 min/week has been shown to be effective. While emerging evidence shows that high-intensity interval training (HIIT) approaches may provide comparable benefit on hepatic steatosis, there does not appear to be an intensity-dependent benefit, as long as the recommended exercise volume is achieved. This dose of exercise is likely to also reduce central adiposity, increase cardiorespiratory fitness and improve cardiometabolic health, irrespective of weight loss. Resistance training should be considered in addition to, and not instead of, aerobic exercise targets. The information in this statement is relevant and appropriate for people living with the condition historically termed non-alcoholic fatty liver disease (NAFLD), regardless of terminology.

**Supplementary Information:**

The online version contains supplementary material available at 10.1007/s40279-023-01918-w.

## Key Points

**Table:** 

Metabolic-associated fatty liver disease (MAFLD) affects one in four adults globally and is linked with type 2 diabetes, cardiovascular disease and lifestyle-related cancers, as well as progressive liver disease and liver cancer.
This position statement collates evidence on the role of exercise in the management of MAFLD including referral pathways, assessment and management priorities, exercise prescription recommendations, action planning and considerations for clinicians working with people with MAFLD. The information in this statement is relevant and appropriate for people living with the condition historically termed non-alcoholic fatty liver disease (NAFLD), regardless of terminology.
The majority of research evidence indicates that 150–240 min per week of at least moderate-intensity aerobic exercise can reduce liver fat by ~ 2–4%, but as little as 135 min/week has been shown to be effective. This dose of exercise is likely to improve central adiposity, cardiorespiratory fitness and cardiometabolic health, irrespective of weight loss.

## Background

Metabolic-associated fatty liver disease (MAFLD), historically known as non-alcoholic fatty liver disease (NAFLD), represents the hepatic manifestation of a multi-system metabolic dysfunction-driven disorder [1]. MAFLD affects at least 25% of people globally and within Australia [2–4]. In addition to increasing the risk of end-stage liver disease and primary liver cancer, MAFLD plays a central role in the development of type 2 diabetes (T2D), cardiovascular disease (CVD) and extrahepatic lifestyle-related cancers [5], reducing life expectancy by 4 years and increasing time spent living with high metabolic burden [6]. In Australia, it is expected that MAFLD cases will increase by 25% between 2019 and 2030 to over 7 million cases [4], considerably elevating the related disease burden. Globally, from 1991 to 2019, MAFLD increased from 22 to 37%, with an annual increase of 0.7% [7].

Given its strong association with obesity, and in the absence of pharmacological agents approved for the long-term management of MAFLD, lifestyle modifications with dietary changes and increased physical activity/exercise remain the cornerstone of MAFLD management. Allied health professionals (e.g. dietitians, exercise physiologists, physiotherapists, health psychologists) play an integral role in managing the burden of MAFLD. The aim of this position statement was to synthesise the literature on the role of exercise for the management of MAFLD and to produce evidence-based statements to be used to develop recommendations for exercise prescription. Evidence to facilitate the translation, implementation and uptake of these recommendations was also reviewed.

### Definitions of Metabolic-Associated Fatty Liver Disease (MAFLD)

**Fig. 1:**
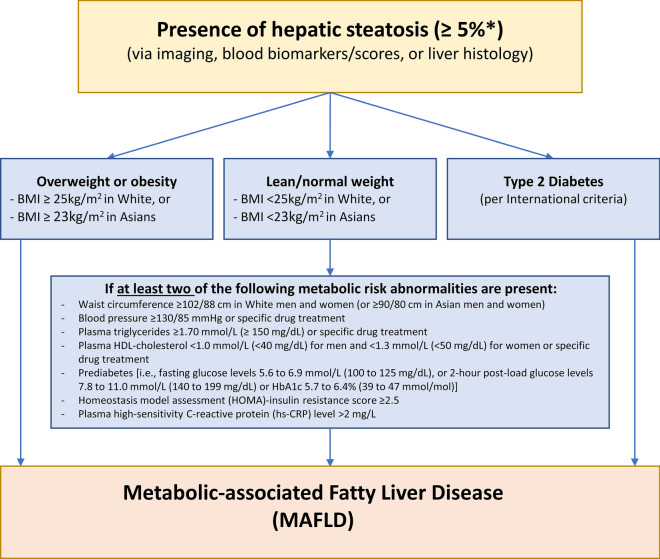
Schematic definition of MAFLD. Adapted from Eslam 2020 [1], with permission. *BMI* body mass index*, HDL-cholesterol* high-density lipoprotein-cholesterol, *HbA1c* glycated haemoglobin, *HOMA* homeostatic model assessment, *hs-CRP* high-sensitivity C-reactive protein. *Relates to liver histology from liver biopsy, with ≥ 5% referring to the proportion of hepatocytes containing visible intracellular lipid droplets. Other modalities (imaging, blood biomarkers/scores) have assigned thresholds to detect steatosis at ≥ 5%

Until recently, MAFLD was known as non-alcoholic fatty liver disease (NAFLD) and was diagnosed if there was evidence of steatosis in ≥ 5% of hepatocytes and the exclusion of other chronic liver diseases and ‘excess’ alcohol intake. NAFLD was further dichotomised into NAFL (referring to simple steatosis) and non-alcoholic steatohepatitis (NASH, referring to the more progressive condition characterised by hepatocyte ballooning, inflammation and varying degrees of fibrosis). Concerns with this terminology included stigma relating to the term ‘alcohol’ and the reprioritisation of disease significance with the term ‘non’. Given the metabolic dysfunction underlying the pathophysiology the term ‘MAFLD’ has been proposed and increasingly used, although a global consensus has not been reached at the time of this publication. MAFLD is defined by the presence of hepatic steatosis in the setting of metabolic dysfunction characterised by having overweight/obesity or T2D, or specific features of metabolic dysregulation [1] (Fig. 1).

MAFLD can coexist with any liver disease (including alcohol related liver disease) and disease severity is further categorised by grade of activity and stage of fibrosis. Importantly for this position statement, many of the citations and reported studies have used the term NAFLD. There is marked overlap between NAFLD as previously defined and MAFLD [1, 8] with high overall concordance of the two definitions (Cohen’s kappa of up to 0.92) [9]. For consistency throughout the statement, we have used the term MAFLD with appropriate clarifications where necessary.

### Development and Clinical Impacts of MAFLD

#### Development

MAFLD develops when there is dysfunction in hepatic fuel utilisation resulting in excess storage of fat (as intrahepatic triglyceride) in the liver (hepatic steatosis) and reduced clearance [via oxidation or repackaging as very low-density lipoprotein (VLDL)-cholesterol]. Hepatic steatosis is a cause and a consequence of insulin resistance [10–12]. Increased free fatty acid (FFA) delivery to the liver primarily arises (59%) from insulin-resistant adipose tissue [13] [especially visceral adipose tissue (VAT) that delivers FFAs directly to the liver via the portal vein [10]]. Additionally, de novo lipogenesis, accounting for a further 26% of FFA flux, uses excess substrate from glucose metabolism (glycerol 3 phosphate), exacerbated by insulin resistance in skeletal muscles, to form intrahepatic triglyceride. Dietary fats, which deliver FFA from the gut via chylomicrons, account for the remaining 15% [13]. Within the liver, these FFAs are excreted by VLDL, oxidised through hepatic β-oxidation, or are synthesised to triglyceride for storage.

#### Clinical Impacts

MAFLD leads to a range of liver-related and extra-hepatic clinical consequences. Obesity and insulin resistance are the key pathophysiological drivers for both liver-related and extra-hepatic disease severity, underpinned by genetic predisposition and/or disruption to the microbiome [14]. People with MAFLD have increased overall mortality, with a 34% higher death rate observed over 7.5 years compared with age- and sex-matched individuals in the general population [15]. The primary causes of death in MAFLD are non-liver related, with CVD and lifestyle-related extra-hepatic cancers accounting for 37% and 21% of deaths, respectively [16]. Approximately 30% of people with MAFLD will develop metabolic-associated steatohepatitis (characterised by hepatocyte injury and death with associated inflammation and varying degrees of fibrosis, historically called non-alcoholic steatohepatitis/NASH); a burgeoning indication for liver transplantation [17, 18]. However, unlike the high incidence of progression from MAFLD to metabolic-associated steatohepatitis, the onset of cirrhosis is relatively low with slow progression (~ 3% in 15 years) [19]. People with histological evidence of steatohepatitis have an increased liver-related mortality rate that is dose-dependent based on the severity of liver fibrosis [20].

### Definitions of Physical Activity, Exercise and Sedentary Behaviour

Physical activity is defined as bodily movement that increases the metabolic rate and can be categorised in relation to the metabolic demands of the activity termed ‘metabolic equivalents’ or METs. Exercise is considered as planned and structured physical activity, generally with a goal to improve or maintain health, wellbeing and/or performance. Exercise prescription centres on the manipulation of programming variables which include the mode of exercise, the frequency (number of sessions per week) of the exercise bouts, the duration (time of the individual exercise bout) and the intensity (the physiological effort/ energy demands of activity). The volume of exercise encompasses the total energy expended in kilojoules per exercise bout or per week, and is a function of the intensity, frequency and duration of weekly exercise. While there are different intensity domain cut points across different professional bodies, intensity is generally framed as ‘light’, ‘moderate’ and ‘vigorous’, and described by approach (e.g. continuous, interval) and modality (e.g. aerobic, anaerobic, resistance). Additionally, sedentary behaviour is classified as activities involving sitting or lying/reclined that have a low energy requirement and little additional movement [21]. Further guidance on exercise prescription variables for both aerobic and resistance training and definitions of intensity domains can be sought elsewhere [21, 22]. The focus of this position paper is on the health effects of exercise on the pathophysiological features of MAFLD, with a specific lens on those that are pertinent to the exercise professional.

### Objectives of the Management of People with MAFLD

#### Clinical Presentation

Most people with MAFLD do not present with specific symptoms; MAFLD is often identified incidentally through routine assessment of blood biochemistry [e.g. abnormal liver enzymes: alanine aminotransferase (ALT) and aspartate aminotransferase (AST)] or on imaging performed for another health reason. MAFLD may be suspected based on an individual’s medical history or the presence of central obesity, or on investigations including abdominal imaging (ultrasound or computed tomography) showing features of hepatic steatosis. There are established criteria to diagnose MAFLD (see Fig. 1) [8, 23], but in addition physicians may consider the following:

(i) *An assessment of the severity of liver injury* including the degree of liver fibrosis. This is most often undertaken with multi-modal non-invasive approaches using bloodwork that relies on routine tests (such as a full blood count and standard biochemistry panels for the NAFLD fibrosis score and Fibrosis-4 (FIB-4) algorithms) and/or serum biomarkers [as used for the enhanced liver fibrosis (ELF) test], or assessments of liver stiffness based on ultrasound [such as vibration-controlled transient elastography (Fibroscan™)], shear wave elastography or acoustic radiation force imaging, or other imaging modalities (e.g. magnetic resonance elastography). Where these non-invasive tests suggest a patient has advanced disease, they may be referred to hepatology services for further evaluation including possible liver biopsy.
(ii) *Other co-morbid contributors to liver disease* including hepatitis C and hepatitis B viruses, and hazardous alcohol consumption as these may require management in conjunction with MAFLD.
(iii) *Medication and over the counter preparations (including herbal remedies and traditional medicines), as well as surgical and general health history.*
(iv) *Screening for cardiometabolic health risk and common comorbidities* including personal and family history of CVD and T2D, metabolic syndrome, hyperglycaemia [e.g. elevated glycosylated haemoglobin (HbA1c), impaired fasting glucose], atherogenic dyslipidaemia, hypertension, smoking status, alcohol intake and anthropometry [including body mass index (BMI), weight history and waist circumference].

**Fig. 2:**
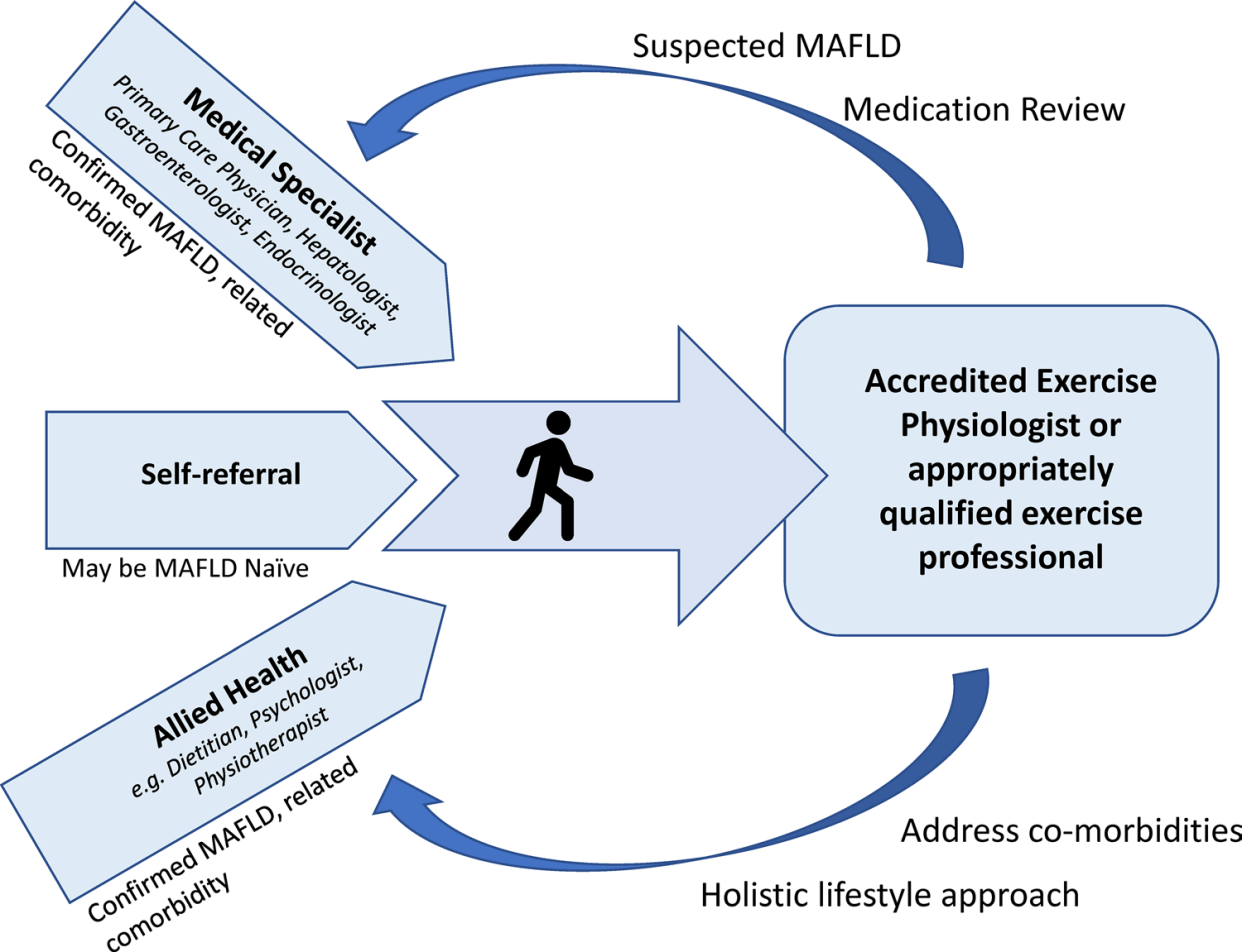
Potential referral and clinical care pathways. *MAFLD* metabolic-associated fatty liver disease

It is common for people to present to exercise services with a chief complaint of T2D, obesity or metabolic syndrome but without a formal specialist review or diagnosis of MAFLD. In these instances, MAFLD may be suspected based on clinical presentation. Given the common underlying pathophysiology, if MAFLD is suspected based on the individual’s clinical history and risk factors (e.g. high waist circumference, T2D, low physical activity), depending on the scope and standards of the local healthcare systems, the exercise professional may facilitate referral to the individual’s primary care physician to establish a diagnosis and determine appropriate care pathways. Potential referral pathways and clinical care team partners are illustrated in Fig. 2.

#### Clinical Assessment and Management Priorities for the Exercise Professional

The management of MAFLD requires a multi-disciplinary approach. From the medical perspective, the overall management priorities for people with MAFLD are centred around two primary goals:

(i) *The resolution of MAFLD and/or prevention of liver disease progression*
(ii) *The prevention of cardiovascular related morbidity and mortality*

The forefront of management for most people with MAFLD is lifestyle therapy, including both dietary modification and regular exercise, with a primary goal of reducing hepatic steatosis, achieving adiposity reduction, managing cardiometabolic comorbidities and preventing lifestyle-related extra-hepatic cancers. Weight loss of 5–10% will result in clinically meaningful health improvements [24]; however, a number of health benefits are likely to be achieved irrespective of significant weight loss. Beyond body weight, a 30% reduction in hepatic steatosis as measured by magnetic-resonance imaging (MRI) is associated with histological improvement and resolution of steatohepatitis [25]. One challenge in assessing the efficacy of therapeutic interventions to improve liver histology is the limited availability of robust measures that can reliably and non-invasively identify clinically meaningful improvements in these parameters. Tools to quantify and monitor liver fat and liver fibro-inflammation are presently available in research settings only. A suite of tools based on common biochemical and anthropometric measures have been proposed as surrogates to imaging methods and liver biopsy [26]. However, these are validated as screening tools only and are not used routinely for assessing change in liver health status, meaning assessing waist and body weight change remains the primary strategy for longitudinal monitoring in response to treatment.

People with MAFLD have lower levels of cardiorespiratory fitness than the general population [27, 28]. Low cardiorespiratory fitness has been reported to be a potent risk factor for MAFLD and is inversely associated with steatohepatitis [29] and liver fibrosis [28], and may predict the degree of steatosis reduction possible with lifestyle intervention [30]. This may explain in part why people with MAFLD frequently report high levels of fatigue, low levels of energy and low exercise-related self-efficacy [31].

Dietary targets include improving diet quality with a focus on heart healthy eating patterns, that are predominantly plant based with abundant daily vegetables, fruit consumption, use of extra virgin olive oil, fish, seafood, legumes and nuts as preferred protein sources and selecting reduced fat dairy and wholegrain bread and cereal options. Reducing intake of alcohol, sugar-sweetened beverages, highly processed foods and processed red meats is also recommended. Practical resources are available elsewhere [32].

Regardless of the presentation or referral pathway, a risk assessment should be undertaken by an appropriately trained exercise professional [e.g. using the Australian Pre-Exercise Screening System (APSS) screening tool].

For the clinical exercise professional, assessment and management priorities for people with known or suspected MAFLD are suggested to include:

(i) *Cardiometabolic risk factors:* Practitioners should assess waist circumference, BMI, blood pressure, family history of heart disease and diabetes, smoking status, alcohol use and physical inactivity. Further clinical assessment, requested by the treating physician, may also include blood lipids and lipoproteins, blood glucose and HbA1c. Scores such as the Atherosclerotic Cardiovascular Disease (ASCVD) score (score calculator freely available online https://www.mdcalc.com/calc/3398/ascvd-atherosclerotic-cardiovascular-disease-2013-risk-calculator-aha-acc) may be used to inform 10 year risk of heart disease or stroke. Management priorities should include reduction in central obesity (as waist circumference), blood pressure and sedentary time, and increasing physical activity. Reducing excess adiposity remains an important part of the clinical management of MAFLD, irrespective of BMI. However, weight loss and/or additional components of metabolic syndrome may also be targets for management depending on clinical presentation. Reductions of 3–5% body weight can improve the cardiometabolic profile [33], reduce liver steatosis [8] and achieve MAFLD remission in people with BMI < 25 kg/m^2^ [34]. Body weight reductions of ≥ 7–10% are recommended for improvements in histological features of MAFLD, especially in people with comorbid MAFLD and overweight/obesity [8, 35]. In people who achieved ≥ 10% weight loss, 90% had steatohepatitis resolution, 81% had fibrosis regression and all improved hepatic steatosis [36].
(ii) *Physical capacity:* Assessment may include physical activity levels and sedentary behaviour, cardiorespiratory fitness, neuromuscular fitness and assessment of any musculoskeletal or orthopaedic limitations (including sarcopenia) that may impact physical and functional capacity. Whilst not necessarily a focus of research to date in MAFLD, it is highly likely that consideration of musculoskeletal health, its assessment and prescription are relevant for many people with MAFLD, when functional capacity and avoidance of muscle loss/sarcopenia are key considerations. Further clinical assessment to establish sarcopenia [37] and frailty via imaging and/or relevant clinical measures such as the short physical performance battery (SPPB) [38] may be clinically appropriate to consider based on individual presentation. While all these outcomes are important for people with MAFLD, priority for assessment and targets for management will depend on individual presentation and intervention targets. Identifying barriers to the uptake and maintenance of physical activity/exercise may also enable personalised exercise programmes (see Sect. 3.3).
(iii) *Comorbidities:* Comorbidities such as metabolic syndrome, pre-diabetes, T2D, obesity, polycystic ovarian syndrome (PCOS), hypertension and depression/mental ill health should be identified, and the broader management of these conditions should be considered in the context of MAFLD management.
(iv) *Patient-important outcome measures:* Practitioners should assess issues particularly relevant to MAFLD such as rating levels of fatigue, energy and exercise-related self-efficacy (e.g. via the Self-Efficacy for Exercise Scale [39]). This may also include sleep quality (e.g. via the Pittsburgh Sleep Quality Index [40]), low-mood, ability to undertake activities of daily living and health-related quality of life (e.g. via the Chronic Liver Disease Questionnaire – Non-Alcoholic Fatty Liver Disease version [41]) or other goals specific to the patient that exercise may address. Patient-important outcome measures could be monitored and assessed using visual analogue scales or the goal attainment scale[42].
(v) *Identification of need for referral onwards* based on clinical opinion. Practitioners should ask about:
- Diet (in accordance with national healthy eating guidelines) with referral to a dietitian if indicated. Referral is indicated if the patient’s needs extend beyond general healthy eating advice, if they have complications due to MAFLD (notably liver cirrhosis, see Sect. 4.3), have additional comorbidities, have specific nutritional composition questions and/or request extra specialist support.
- Emotional, social and cognitive functioning with referral to a psychologist or behavioural counsellor if indicated.
- Medication use to determine whether the individual is taking medication (e.g. for blood pressure, T2D, cholesterol) and if not, referring to their primary care physician for medical review if indicated based on clinical assessment. Medications may also need modifying by the primary care physician as the patient starts regular exercise or significantly changes their exercise programme (e.g. to reduce antihypertensives, oral hypoglycaemics or insulin).
- If MAFLD is suspected, referral back to the primary care physician to establish a pathway for diagnosis, assessment of disease severity and additional care pathways if required.

## Section 2: Evidence for the Role of Exercise in the Management of MAFLD

**Fig. 3:**
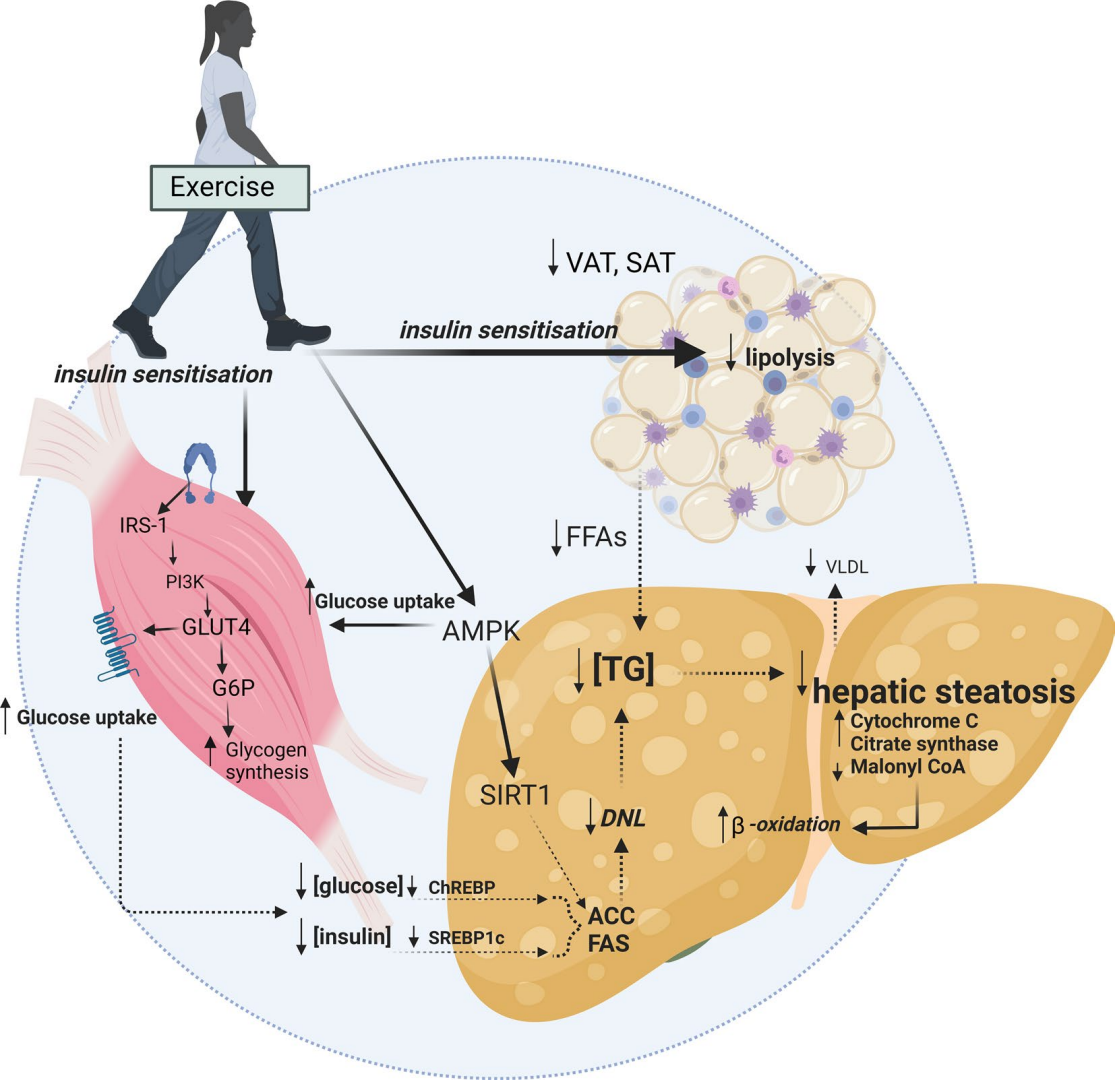
Putative mechanisms for the effects of exercise on reducing hepatic steatosis Solid lines indicate enhanced mechanisms (e.g. insulin sensitisation). Dashed lines indicate reduced mechanisms (e.g. reduced concentrations of plasma insulin and glucose, reduced de novo lipogenesis). *VAT* visceral adipose tissue, *SAT* subcutaneous adipose tissue, *FFA* free fatty acid, *TG* triglyceride, *SREBP- 1c* sterol regulatory element binding protein, *ChREBP* carbohydrate responsive element binding protein, *DNL* de novo lipogenesis, *FAS* fatty acid synthase, *ACC* acetyl-coenzyme A carboxylase, *VLDL* very low-density lipoprotein-cholesterol, *AMPK* adenosine monophosphate-activated protein kinase, *SIRT1* sirtuin 1, *IRS-1* insulin receptor substrate 1, *PI3K* phosphatidylinositol-3-kinase, *GLUT4* glucose transporter type 4, *G6P* glucose 6-phosphate. Created with BioRender.com

There is clear and consistent evidence that regular exercise is cardioprotective and has multiple benefits on musculoskeletal function and mental health, irrespective of weight loss [43]. The mechanisms by which exercise modulates hepatic steatosis have been detailed elsewhere [14, 44–46]. Briefly, putative mechanisms centre on altering the flux of free fatty acids (FFA) to and from the liver via changes to substrate metabolism within the muscle, adipose tissue and liver [11, 47, 48]. These may be mediated, in part, by improvements in peripheral insulin sensitivity and glucose uptake which alter liver signalling pathways [i.e. sterol regulatory element binding protein-1c (SREBP-1c) and carbohydrate response element binding protein (ChREBP)] and gene expression [e.g. lipogenic proteins fatty acid synthase (FAS) and acetyl-coenzyme A carboxylase (ACC)]. In rodents, exercise-mediated increases in mitochondrial enzymes (e.g. cytochrome c oxidase, citrate synthase and β-hydroxyacyldehydrogenase) and increases in mitochondrial content and oxidative capacity have been shown [49]. These improvements in liver mitochondrial content and function have been associated with increased β-oxidation, which may prevent the accumulation of metabolic by-products such as ceramides and diacylglycerides that contribute to insulin resistance (Fig. 3). A key signalling pathway for direct liver benefit may be the activation of the metabolic energy sensor adenosine monophosphate-activated protein kinase (AMPK). This pathway plays an important role in regulating metabolism (i.e. increasing fatty acid oxidation and glucose uptake). AMPK activity is reduced in obesity, diabetes and inflammatory states [50] and is increased acutely during and after exercise in mice [51], with indirect evidence suggesting that exercise modulates the AMPK/mTORC1 pathway in people with MAFLD [52]. A recent phase 2a pharmacological trial targeting AMPK activation observed reduction in liver fat and metabolic parameters in some people with MAFLD [53]. Extra-hepatic adaptations to exercise, notably exercise-mediated reductions in VAT, may also reduce the delivery of FFA and to the liver [11].

### Literature Search

A systematic online literature search for systematic reviews with meta-analyses was conducted (by SK) from database inception to June 2023 across seven electronic databases [PubMed, Cochrane Library, Embase (Ovid), CINAHL (Ebsco host), Web of Science and SPORTDiscus]. Search terms included keywords and Medical Subject Headings (MeSH) to find literature involving exercise and liver fat and/or MAFLD populations (see Online Resource 1 for the full list of search terms and specific database strategies). Included reviews were systematic reviews with meta-analyses that were in a MAFLD cohort and/or reported on a liver outcome (e.g. hepatic steatosis or liver biochemistries). The evidence generated by extraction (by SK and AS) and collation of literature via these searches was reviewed and evidence was graded (all authors). Consensus on the content and recommendations of the position statement was reached through an iterative process involving the multi-disciplinary authorship team. Decisions on how evidence-based guidelines could be best translated into clinical practice were developed via evidence review and authors’ professional experiences.

The majority of evidence was from early stage MAFLD, with limited data from people with metabolic-associated steatohepatitis and/or cirrhosis. Five overarching evidence statements were defined, and the strength of each evidence statement was graded based on the National Health and Medical Research Centre (NHMRC) guidelines (Table 1). The evidence grade reflects the degree of certainty based on the overall body of evidence that an effect or association is correct.

**Table 1:** Evidence Statements

Statement or recommendation	Grade
Hepatic steatosis	
Exercise is effective for reducing hepatic steatosis by modest amounts (2–4% absolute reductions). High certainty of evidence	A
Aerobic exercise of at least moderate-intensity results in moderate absolute reductions in hepatic steatosis of ~ 2–4% in adults with MAFLD. High certainty of evidence	A
The benefit of aerobic exercise on reduction in hepatic steatosis may extend to children and adolescents, but because there are fewer studies the certainty of evidence for this is low	C
The efficacy for resistance training on reducing hepatic steatosis is uncertain. There is some evidence that resistance training may reduce hepatic steatosis by a modest amount; however, evidence is mixed possibly because there is significant variability in study methodologies including the resistance training prescription. Low certainty of evidence	C
There is limited evidence for the effect of combined (same session) aerobic and resistance training on hepatic steatosis. No recommendations due to insufficient evidence	Consensus-based recommendation
Emerging evidence suggests that HIIT may be comparable to moderate intensity continuous training for reducing hepatic steatosis; however there is insufficient evidence to make firm recommendations for HIIT. Moderate certainty of evidence	B
There is limited evidence for the effect of SIT or other novel training approaches (e.g. acceleration training, Pilates) on hepatic steatosis. No recommendations due to insufficient evidence	Consensus based recommendation
Liver histology – fibrosis, inflammation, hepatocyte ballooning, NAFLD activity score	
There is minimal evidence for the effect of exercise on histological features of MAFLD or liver disease severity beyond the established benefits of exercise on hepatic steatosis and on general health and wellbeing. No recommendations due to insufficient evidence	D
Liver enzymes	
Aerobic exercise appears effective for improving ALT by a small (6–7 IU/L) amount. Low certainty of evidence	C
The efficacy of resistance training for improving liver enzymes is unclear. No recommendations due to insufficient evidence	D
There is minimal evidence for the effect of HIIT for improving ALT. No recommendations due to insufficient evidence	D
There is limited evidence for the effect of combined exercise training, SIT or other novel training approaches on liver enzymes. No recommendations due to insufficient evidence	Consensus based recommendation
Anthropometrics	
Exercise reduces BMI by a small amount (~ 0.8 kg/m^2^). High certainty of evidence	A
Exercise appears to reduce waist circumference by a modest (~ 1.2 cm) amount. Low to moderate certainty of evidence	C
Aerobic exercise is effective for improving BMI by a small (~ 0.85–0.97 kg/m^2^) amount. High certainty of evidence	A
There is limited evidence for the effect of combined exercise training, resistance training, HIIT, SIT or other novel training approaches on body weight or waist circumference. No recommendations due to insufficient evidence	Consensus based recommendation
Aerobic exercise may improve VAT in people with MAFLD; however, evidence in populations with MAFLD is lacking. Low certainty of evidence	D
Comorbidities	
Aerobic exercise improves cardiorespiratory fitness by a clinically meaningful (~ 3.5–8.0 ml/kg/min) amount in people with MAFLD. High certainty of evidence	A
Exercise appears to improve total cholesterol and LDL-cholesterol in people with MAFLD. Moderate certainty of evidence	B
There is minimal evidence for the effect of exercise on other cardiometabolic risk factors or comorbidities associated with MAFLD including glycaemic control, vascular health and health-related quality of life. No recommendations due to insufficient evidence	D

Grade category description: Evidence-based recommendations (A-D): **A**, body of evidence can be trusted to guide practice; **B**, body of evidence can be trusted to guide practice in most situations; **C**, body of evidence provides some support for recommendation, but care should be taken in its application; **D**, the body of evidence is weak and the recommendation must be applied with caution. Consensus-based recommendation, recommendation based on clinical opinion and expertise as insufficient evidence is available. *MAFLD* metabolic-associated fatty liver disease, *HIIT* high-intensity interval training, *SIT* sprint interval training, *ALT* alanine aminotransferase, *AST* aspartate aminotransferase, *BMI* body mass index, *LDL* low-density lipoprotein, *VAT* visceral adipose tissue

### Evidence for the Benefits of Exercise on Hepatic Steatosis

As at June 2023, 25 systematic reviews with meta-analyses had examined the efficacy of exercise for reducing hepatic steatosis [54–77]. Reviews predominantly included randomised controlled trials (RCTs) in adults [54, 55, 58, 59, 61, 62, 64, 67, 68, 70–74, 76–81], children or adolescents [59, 63], or both [66], with overweight or obesity [58, 66, 71] and/or confirmed MAFLD [54, 64, 65, 69, 72–77, 79–82] or related cardiometabolic diseases [68]. Most employed aerobic exercise interventions with substantially fewer examining resistance training, interval training or combined aerobic and resistance training interventions. Comparator groups were either usual care or non-exercise controls. Some studies included a combined exercise plus diet arm with a diet-only comparison arm and were included only if the dietary interventions were the same in both groups. Hepatic steatosis outcomes were generally examined via magnetic resonance techniques, computed tomography approaches or liver biopsy [58, 59, 63, 67–69], but earlier reviews also included liver ultrasound.

Collectively, studies have demonstrated a benefit of exercise for reducing hepatic steatosis. In the most current of these meta-analyses, medium pooled effect sizes were observed favouring exercise [54, 66] reflecting absolute reductions in hepatic steatosis between − 2.40% (95% CI − 3.13 to − 1.66%) [55] and as much as − 5.1% (− 8.1 to − 3.6%) [77] for exercise compared with usual care/no exercise comparator. Larger effects were apparent in populations with established MAFLD [60, 66] and in those with higher baseline BMI [66]. While fewer studies are available in children and adolescents, the observed effect for the benefit on hepatic steatosis appears consistent with that for adults [66] with absolute reductions of − 2.10% (95% CI − 3.25%, − 0.95%) [63]. Collectively, no moderating effects were observed for exercise training variables (i.e. frequency, session duration, study duration or volume) on change in hepatic steatosis [66, 70, 83]. It is unclear precisely how much change in steatosis is due to exercise itself versus weight loss, but weight loss is a key predictor of steatosis reduction [84]. However, it is also clear that significant and meaningful reductions are achievable in the absence of weight loss, or with weight loss that is not considered clinically meaningful (i.e. < 3–5%) [84].

The clinical impact of a 2–4% absolute reduction in liver fat is not well established given the lack of long-term data regarding change in hepatic steatosis and hard clinical outcomes (e.g. mortality, cardiovascular events). However, the effect sizes are comparable to those observed for most pharmacological agents [65] and larger than Mediterranean-style dietary interventions without energy restriction [85, 86]. Notably, a ≥ 30% relative decrease from baseline in hepatic steatosis was associated with higher likelihood of histological change and resolution of steatohepatitis [25]. Pooled analyses of exercise trials compared with non-exercise control show that exercise training participants with MAFLD are 3.5 times more likely to achieve this threshold than patients receiving usual care [77]. In an individual with an absolute liver fat of 13%, a 2–4% absolute reduction would reflect a 15–30% relative reduction which may be clinically meaningful for both cardiometabolic and liver-related disease risk. However, it is important to note that there is no evidence to date conferring that an improvement in these surrogate parameters directly contributes to a reduction in cardiovascular disease mortality.

#### Aerobic Exercise

Sabag et al. 2022 collated evidence (to December 2020) on aerobic exercise modalities compared with non-exercising comparators on hepatic steatosis, quantified by magnetic resonance methods in adults. A medium pooled effect for moderate-intensity continuous training compared with control was observed, translating to an absolute reduction in hepatic steatosis of − 3.14% (95% CI − 4.45%, − 1.82%) [83]. Previous reviews have reported similar magnitude of effects [64, 70].

Effective doses for moderate-intensity aerobic exercise have ranged from three to seven sessions per week (mode: three/week) for 4–52 weeks (mode: 12 weeks) at moderate–vigorous intensity (based predominantly on percentage heart rate maximum or percentage of peak oxygen consumption ($\dot{V}$O_2_peak) or median effective metabolic equivalents (METs) per session of 4.8 METs [87]). Exercise volume ranged from 135 min/week [88, 89] to 240 min/week [89], with a median effective duration reported as 40 min per session [87]. Aerobic exercise modalities included predominantly walking, treadmill and stationary cycling, with some studies reporting ‘various’ modalities including rowing and elliptical training. Continuous vigorous-intensity aerobic exercise was less commonly employed with only two studies prescribing a ‘vigorous’ intensity arm on 3 days per week for 8 weeks [89] and 5 days per week for 6 months [90],with a mean reduction in hepatic steatosis of 2.4% and 5.0%, respectively. Keating et al. found a reduction in hepatic steatosis compared with control regardless of intensity or volume including (i) 60 min, 4 days/week, 50% ${\dot{\text{V}}\text{O}}_{{2}}$peak; (ii) 45 min, 3 days/week, 70% ${\dot{\text{V}}\text{O}}_{{2}}$peak; and (iii) 45 min, 3 days/week, 50% ${\dot{\text{V}}\text{O}}_{{2}}$peak [89]. Similarly, Zhang et al*.* found no differences in magnitude of reduction in hepatic steatosis at 6 months following either moderate- or vigorous-intensity exercise training [90]. These findings were corroborated by a meta-regression demonstrating no association between either total exercise volume in minutes per week or energy expenditure (Kcal) per week and reductions in hepatic steatosis across 18 RCTs [67]. Collectively, this evidence demonstrates that the apparent minimum effective dose of exercise to improve hepatic steatosis is 135 min of moderate-intensity aerobic activity per week with no additional benefit on hepatic steatosis from increasing the intensity of exercise.

#### Resistance Training

There is a relative paucity of evidence on the effect of resistance training on hepatic steatosis. There have been several intervention studies utilising different resistance training methodologies; e.g. ‘traditional’ progressive resistance training with varying volumes and intensities in adults [91–96] and adolescents [97–99], or as circuit training [100–102]. There is no consistent evidence, with an approximately equal number of studies demonstrating benefit [91, 94, 95, 97, 100, 102] or no benefit [92, 93, 98, 99, 101] of resistance training on hepatic steatosis. There are no clear signals for beneficial effects of specific resistance training approaches. Potentially, people with more severe metabolic derangements (e.g. established T2D) and greater baseline levels of hepatic steatosis may achieve reductions in hepatic steatosis with resistance training in isolation [55]. There have been no comparisons of resistance training dose or the effectiveness and safety of resistance training protocols delivered beyond the clinical/laboratory-based settings in people with MAFLD.

#### Combined Aerobic Exercise + Resistance Training

There is very little evidence for the efficacy of combined aerobic exercise plus resistance training for reducing hepatic steatosis. Combined aerobic and resistance training 3 days per week for 45–60 min led to reductions in hepatic steatosis of ~ 2% at 12 weeks [103] and ~ 10% at 16 weeks [104].

#### High-Intensity Interval Training

High-intensity interval training (HIIT) is characterised by high-intensity bouts of exercise interspersed with passive or low-intensity rest periods [105]. Typical HIIT approaches involve completing one to ten bouts of high-intensity exercise lasting between 1 and 4 min, interspersed with 30 s to 3 min rest periods. Emerging evidence suggests that HIIT may be beneficial for reducing hepatic steatosis [62, 83], with one meta-analysis reporting a medium pooled effect for HIIT compared with control translating to absolute reductions of − 2.85% (95% CI − 0.95%, − 0.23%) [67]. HIIT may be at least comparable to moderate-intensity continuous training (MICT) for improving hepatic steatosis; however, the certainty of evidence regarding comparability to MICT is low due to limited evidence [62, 67]. HIIT approaches which have been shown to improve steatosis involved one to five intervals of high-intensity aerobic exercise lasting 2–4 min with 2–3 min rest between intervals.

#### Sprint Interval Training and Other Training Approaches

The effect of sprint interval training (SIT), characterised by ‘all-out’ or supramaximal (> 100% V̇O_2_max) bouts lasting between 8 and 30 s, on hepatic steatosis has been investigated in a 2 week study in people with, or at risk of, T2D [106]. SIT resulted in comparable absolute reductions in hepatic steatosis (~ 3%) to moderate-intensity continuous training in those with impaired glucose tolerance.

Research is emerging regarding other novel training approaches such as whole-body vibration (including acceleration training) [107–109] and hybrid training (involving antagonist muscle electrical stimulation during agonist contraction) [110–112] and Pilates [113], with very low certainty of evidence for benefit [107, 108, 111, 112] or no benefit [109, 110] on hepatic steatosis.

### Evidence for the Benefits of Exercise on Liver Histology: Fibrosis, Lobular Inflammation, Hepatocyte Ballooning and NAFLD Activity Score

There are limited data for the efficacy of exercise for improvements in the histological features of MAFLD beyond steatosis (i.e. fibrosis, lobular inflammation, hepatocyte injury and NAFLD activity score). A cross-sectional analysis of 813 individuals with biopsy confirmed MAFLD showed that those who self-reported undertaking vigorous intensity exercise (75 min/week) had a reduced likelihood of steatohepatitis. Only those who self-reported high volumes (150 min/week) of vigorous intensity exercise had a reduced likelihood of advanced fibrosis [114]. Analyses of the UK Biobank population-cohort (*n* = 840) demonstrated that device-measured moderate–vigorous physical activity was inversely associated with hepatic fibro-inflammation (quantified using multi-parametric MRI), even in those without pre-existing MAFLD, and independent of sociodemographic and other significant lifestyle factors, including diet [115]. While these data suggest that exercise is protective, whether exercise is beneficial for improving histological aspects of established MAFLD is unclear, with evidence from RCTs lacking. To date, just three trials have undertaken paired liver biopsy across a total of 34 exercising participants with mixed findings [101, 116, 117]. A recent small study observed a one-stage regression in fibrosis in 58% of participants, and a one-stage regression in hepatocyte ballooning in 67% of participants (*n* = 12) following 12 weeks of moderate-to-vigorous [40–75% heart rate reserve (HRR)] aerobic exercise intervention on 3–5 days per week; however, there was no control group for comparison and there was heterogeneity in the histological phenotype of participants [117].

### Evidence for the Benefits of Exercise on Liver Enzymes

Liver enzymes ALT and AST are commonly measured as indicators of liver injury; however, there is significant biological variation in repeat measures of ALT and AST, and AST of muscle origin may increase dramatically following acute exercise [118]. Coupled with poor correlation with liver histology [119], these liver enzymes should not be considered as a good surrogate for hepatic steatosis or MAFLD severity in isolation. To date, 21 meta-analyses and two network analyses have examined the efficacy of exercise for improving ALT [54, 57, 60, 61, 64–66, 70, 72, 74–76, 80–82, 120–126]. Nineteen of these have also examined AST [54, 57, 60, 64–66, 70, 72, 74–76, 80–82, 120–123, 126], with mixed findings for the benefits of exercise on liver enzyme reduction. Based on the most current meta-analyses, low-to-medium pooled effect sizes demonstrating a benefit of exercise on ALT have been observed [54, 74–76, 80, 81, 120, 121, 126], showing absolute differences of 6.66 IU/L (95% CI 3.27, 10.04 IU/L) [126] favouring exercise versus control. Low-to-medium pooled effect sizes favouring exercise have also been observed for AST [74, 75, 81, 120, 121, 126], showing absolute differences of 3.14 IU/L (95% CI 0.35, 5.93 IU/L) [116] favouring exercise versus control [126]. The majority of evidence for the benefit of exercise on ALT is from aerobic exercise training studies [54, 75, 120, 126], with no effect observed with resistance training in isolation [121, 126] or when combined with aerobic training [54]. Two recent network meta-analyses found no benefit for any modality of exercise (including aerobic, resistance, combined aerobic plus resistance or HIIT) on ALT or AST [122, 123].

It is unclear whether these statistically significant but modest improvements in liver enzymes are clinically meaningful in isolation. Notably, a clinically significant change in ALT is ≥ 17 IU/ml which represents a cut off that surrogates for fibrosis change, and there are no data to inform the clinical impact of smaller changes in liver enzymes, which are likely to be influenced by natural variability [127].

### Evidence for the Benefits of Exercise on Body Weight and Body Composition

The influence of exercise on body weight [128, 129], waist circumference [130] and body composition [131, 132] has been well described elsewhere. Specific to the included reviews (in MAFLD populations), the available evidence appears to demonstrate that regular exercise improves BMI by a small amount in people with MAFLD or related populations reporting liver outcomes [55, 64, 70–74, 121, 123, 126]. A recent meta-analysis that pooled 13 studies, showed a small but significant effect favouring exercise versus control [− 0.78 kg/m^2^ (95% CI − 1.07, − 0.48 kg/m^2^)] [121], with absolute non-significant reductions of − 2.43 kg (95% CI − 4.99, 0.14 kg) between exercise and control [72]. The magnitude of effect of exercise alone on body weight change (i.e. without concomitant dietary modification) is minimal with weight change on average less than 2–3 kg.

Three reviews have examined the efficacy of exercise for reducing waist circumference in people with MAFLD, with these showing moderate pooled effect sizes favouring exercise [60, 65, 73] that translate to reductions of − 1.24 cm (95% CI − 2.15, − 0.34 cm) [65]. This magnitude of reduction is likely clinically meaningful given a 1 cm increase in waist circumference is associated with an approximately 2% increase in risk of CVD [74, 133]. Only one study [134] has pooled data from four studies to examine the effect of exercise on lean body mass, finding no significant effect compared with control [1.01 kg (95% CI − 1.78 to 3.8 kg)].

Most evidence for the efficacy of exercise for reducing BMI in people with MAFLD is based on aerobic exercise interventions. Mean differences of up to − 0.97 kg/m^2^ (95% CI − 1.40, − 0.55 kg/m^2^) [70, 121] have been reported for aerobic exercise studies compared with control. Collectively, for the small reduction of body mass, effective studies have prescribed interventions of 150–240 min per week of moderate intensity aerobic exercise for 30–60 min three to five times per week for 12 weeks to 12 months [89, 90, 135, 136], or 60 min/week of moderate–vigorous aerobic exercise (60–85% ${\dot{\text{V}}\text{O}}_{{2}}$peak) for 6 weeks [137]. Similar doses of aerobic exercise have been shown to be efficacious for reduction in waist circumference in MAFLD and related populations [89, 90, 135–137].

There are limited data to inform the efficacy of aerobic exercise for the modulation of VAT in people with MAFLD. Studies have reported mixed findings [89–91, 97, 98, 103, 135, 136, 138–142] due in part to heterogeneity in exercise prescription and VAT analysis methodology. A recent meta-analyses pooled data from six studies and reported a mean difference favouring exercise training of − 8.30 cm^2^ (− 11.59 to − 5.00 cm^2^) [74]. However, in populations with overweight or obesity, in whom MAFLD is present in up to 80%, aerobic exercise has been shown to reduce VAT [130] and it is therefore likely that these benefits are generalisable to people with MAFLD. In adults, several studies have demonstrated both significant or near significant between-group [89–91, 103, 135] and within-group [138, 139] effects for reduction in VAT in studies employing predominantly aerobic exercise between 8 weeks and 6 months durations, while others have reported no significant changes [136, 140, 141]. In a large study by Zhang et al., only higher volume vigorous-intensity exercise significantly reduced VAT compared with control, despite both moderate and vigorous exercise reducing hepatic steatosis [90].

### Extra-Hepatic Benefits of Exercise in Patients with MAFLD

The benefit of regular exercise for reducing risk of some extra-hepatic, lifestyle-related cancers is well established [143]. The profound benefits of exercise on cardiometabolic risk [144, 145], vascular health [146] and cardiorespiratory fitness [144, 147] in a broad range of clinical populations [148] are detailed elsewhere. While emerging evidence from small RCTs suggests these extra-hepatic benefits are likely to be expected with exercise intervention in people with MAFLD, data are relatively limited.

#### Cardiorespiratory Fitness

Evidence from meta-analyses [55, 72, 75, 82, 134] shows mean improvements in cardiorespiratory fitness of between 3.61 ml/kg/min (95% CI, 2.27, 4.94 ml/kg/min) and 8.25 ml/kg/min (95% CI, 5.27, 11.24 ml/kg/min) for exercise compared with control. These studies have largely involved supervised moderate-intensity continuous aerobic training in controlled laboratory settings in people with MAFLD. There have been indications that change in hepatic steatosis is strongly and inversely associated with change in ${\dot{\text{V}}\text{O}}_{{2}}$peak, to a greater degree than change in body weight (*r* =  − 0.880 and *r* = 0.666 respectively) [55]. Every 1 ml/kg/min increase in V̇O_2_peak is linked with a 0.87% reduction in liver fat (95% CI − 1.5%, − 0.2%) [55]. In the general population a 3.5 ml/kg/min increase in cardiorespiratory fitness is associated with a 13% and 15% reduction in all-cause and CVD-related mortality, respectively [149], and is therefore likely clinically meaningful for those with MAFLD, although direct evidence of this is limited. Notably, this magnitude of change has been observed in as little as 4 weeks of moderate to vigorous intensity aerobic exercise for 135 min/week [88].

Effective doses of exercise have primarily involved moderate-to-vigorous intensity aerobic exercise on 3–5 days per week for 30–60 min per session over 4 weeks to 8.6 months [88, 89, 136, 150, 151]. While exercise-induced improvements in steatosis appear to be associated with improvements in cardiorespiratory fitness, the efficacy of HIIT for improving both measures (fitness and steatosis) concomitantly has not been established. Of the three studies to report both outcomes [138, 141, 152], all reported an improvement in hepatic steatosis but only two reported improvements in cardiorespiratory fitness [mean difference ~ 2.2 ml/kg/min (95% CI − 0.96, 5.39 ml/kg/min) compared with control] [138, 152] using one to three 4 min intervals at 80–90% V̇O_2_peak. Based on comprehensive evidence in other related populations [153, 154], HIIT is likely to provide substantial improvements in cardiorespiratory fitness; however, data are lacking and therefore benefit cannot be assumed.

#### Cardiometabolic Risk Variables and Health-Related Quality of Life

Various systematic reviews with meta-analyses have reported pooled analyses for several cardiometabolic outcomes across a broad range of exercise prescriptions. The majority of evidence supports the benefits of exercise on total cholesterol [64, 71, 74, 75, 121, 124, 125], potentially driven by benefits on LDL-cholesterol [54, 64, 65, 75, 79, 82, 121, 124, 125]. While one meta-analysis demonstrated within-group benefits for exercise on hepatic insulin sensitivity [69], the efficacy for improving glycaemic control [54, 65, 73, 75, 79, 80] and the Homeostatic Model Assessment for Insulin Resistance (HOMA-IR) [25, 50, 54, 59, 60, 66, 67, 70, 76, 123] in people with MAFLD is inconsistent. Small laboratory-based aerobic exercise trials have shown a benefit on endothelial function [136], peripheral insulin sensitivity [142] and novel risk factors such as thrombotic biomarker plasminogen activator inhibitor 1 (PAI-1) [139]. While health-related quality of life is an important outcome across all chronic disease groups, there is limited experimental evidence available that this improves with exercise in MAFLD [72, 134]. Other newer emerging studies demonstrate improvement in patient-important outcomes such as pain interference and social roles [139].

## Recommendations for Exercise Prescription

An overview of exercise prescription recommendations to achieve specific exercise-modifiable goals for people with MAFLD is presented in Table 2. It is important for the exercise professional to identify and acknowledge the potential for low-physical capacity and follow the framework underpinning the principles of exercise prescription, namely an individually tailored and progressive approach. Additionally, prudent advice around minimising sedentary behaviour and maximising the opportunity for movements should complement formal exercise prescription. All people with MAFLD seeking to reduce hepatic steatosis should progressively target a minimum of 135 min of at least moderate-intensity aerobic exercise per week and ideally aim for 150–240 min per week. There is no conclusive evidence to support an intensity-dependent benefit for reducing steatosis so long as the recommended volume is achieved. This prescription is also beneficial for improvements in cardiorespiratory fitness and likely other cardiometabolic risk factors, including VAT, although Grade A level evidence is specifically lacking in MAFLD. HIIT approaches may provide comparable benefits on hepatic steatosis and be prescribed alone or in conjunction with moderate-intensity continuous training depending on individual preference, risk stratification and capabilities. However, it is important to highlight that clinical guidelines for the safe delivery of HIIT in people with chronic disease emphasise that medical screening, cardiopulmonary evaluation, modest workload progression and ongoing monitoring of health and medication status are necessary [155], and that high-intensity and sprint interval training may be contraindicated in some people with MAFLD. For those with no contraindications who seek variation in their training programme, or prefer HIIT, HIIT may be offered as an alternative. However, HIIT should not be touted as superior in view of the current evidence. Improvements in total and central adiposity may require higher volumes of aerobic exercise although data are limited in MAFLD.

**Table 2:** Exercise prescription recommendations for MAFLD

Management goal		Aerobic only	Resistance only	Practical considerations
	Reduction in hepatic steatosis	**Moderate to vigorous**^**a**^ intensity aerobic (e.g. brisk walking, jogging, cycling)^b^ exercise for at least **135 min per week** of moderate intensity across **3–5 days per week**; ideally progressing to **150–240 min per week.** *Strong evidence* And/or High-intensity interval training (HIIT) involving **1–5 high-intensity intervals**^**a**^ of **2–4 min** interspersed with **2–3 min lower-intensity recovery**^**c**^ between intervals on **3–5 days per week.** *Moderate evidence*	Insufficient evidence	Aerobic exercise and/or HIIT should be prioritised for the management of hepatic steatosis The benefits of resistance training in isolation are unclear. Resistance training should be included in addition to the recommended volume of aerobic exercise but not instead of it. Clinicians aiming to prescribe resistance training to individuals with cardiometabolic disorders may find appropriate resources elsewhere [137, 138]
	Reduction in central adiposity (waist circumference as a surrogate for VAT)	**Moderate to vigorous**^**a**^ intensity aerobic (e.g. brisk walking, jogging, cycling)^b^ exercise for at least **150–240 min per week** of moderate intensity across **3–5 days per week.** *Weak–moderate evidence*	Insufficient evidence	As little as 60 min of weekly vigorous intensity activity may provide equivalent benefit Practical considerations for resistance training as per hepatic steatosis
	Improvement in cardiorespiratory fitness	**Moderate to vigorous**^**a**^ intensity aerobic (e.g. brisk walking, jogging, cycling)^b^ exercise for at least **135 min per week** of moderate intensity across **3–5 days per week** *Strong evidence*	Insufficient evidence	Emerging evidence suggests HIIT may equally improve cardiorespiratory fitness in people with MAFLD
	Weight loss	**Moderate to vigorous**^**a**^ intensity aerobic (e.g. brisk walking, jogging, cycling)^b^ exercise for at least **150–240 min per week** of moderate intensity across **3–5 days per week.*** Strong evidence*	Insufficient evidence	Important to appropriately communicate the modest magnitude of weight loss (~ 2–3 kg or ~ 1.5%) expected with exercise intervention of this volume in isolation Practical considerations for resistance training as per hepatic steatosis

*MAFLD* metabolic-associated fatty liver disease, *HIIT* high-intensity interval training, *VAT* visceral adipose tissue, *VO*_*2*_*R* rate of oxygen consumption reserve, *HRR* heart rate reserve, *RPE* rating of perceived exertion, *VO*_*2*_*peak* peak rate of oxygen consumption

^a^Recommended intensity based on the research papers on which the evidence was based. Moderate: 40–60% of ${\dot{\text{V}}\text{O}}_{{2}}$R or HRR, or 12–13 RPE; vigorous: 60–84% of ${\dot{\text{V}}\text{O}}_{{2}}$R or HRR, or 14–16 RPE; high intensity: ≥ 85–100% ${\dot{\text{V}}\text{O}}_{{2}}$peak or ≥ 15 RPE

^b^There is no evidence for comparative efficacy of these aerobic exercise training modalities

^c^No studies have used passive recovery periods

Despite no clear evidence for beneficial effects of resistance training on hepatic steatosis and central adiposity, progressive resistance training is necessary to maintain/limit losses in lean muscle mass during weight loss and elicits a range of other functional and cardiometabolic benefits that may warrant its inclusion in addition to aerobic exercise training for people with MAFLD. Practitioners may find relevant recommendations for resistance training prescription elsewhere [156, 157].

Except for the recommendations regarding HIIT for which there are no studies, these recommendations may also benefit people with cirrhosis, although further clinical considerations are applicable (see Sect. 4.3 and further extensive review by Tandon and co-workers [158]).

### Limitations Based on Available Evidence

The prevailing limitation in the available evidence is the lack of data to inform the effects of exercise on histological endpoints (i.e. liver fibrosis, improvement in NAFLD activity score, steatohepatitis resolution) that inform liver disease severity, due to the challenges, ethical considerations and recruitment barriers relating to repeated liver biopsy. However, the emergence of validated non-invasive alternatives to biopsy such as multi-parametric MRI [159] may facilitate our understanding of the efficacy of exercise on liver fibro-inflammation. Whether exercise in isolation resolves MAFLD and the efficacy of exercise for reducing cardiovascular and liver disease mortality are also yet to be established.

Regarding exercise prescription, the utility of progressive resistance training for improving hepatic steatosis and other cardiometabolic variables in MAFLD is unclear. While emerging evidence suggests that HIIT may be beneficial for reducing hepatic steatosis and improving cardiorespiratory fitness, there is considerable variability in HIIT protocols which limits the ability to recommend effective doses. Moreover, sustainability of HIIT outside supervised settings has yet to be demonstrated. There are no data to suggest a benefit of SIT, although given the number and nature of comorbidities often present in people with MAFLD it is unknown whether the supra-maximal efforts required to drive the known peripheral adaptations would be attained or sustained, and its safety beyond controlled supervised laboratory settings is unknown. Similarly, novel approaches including whole body vibration, acceleration and hybrid training involving muscle stimulation are unlikely to be broadly translatable, especially in home-based programmes, given the sophisticated equipment required to undertake these approaches. Moreover, despite a broad literature for other chronic conditions, evidence to inform exercise prescription to improve non-steatosis outcomes such as body weight, cardiometabolic risk factors, cardiorespiratory fitness and health-related quality of life in people with MAFLD is relatively limited.

Collectively, small sample sizes, methodological heterogeneity, population heterogeneity (e.g. MAFLD versus steatohepatitis versus cirrhosis) and environmental heterogeneity (e.g. gym versus community locations) limit the ability to examine the relative importance of prescription variables, except for intensity-independent benefits of aerobic exercise on hepatic steatosis. As most interventions have been conducted in supervised clinical or laboratory settings, the translatability of effective exercise interventions into ‘real-world’, self-directed settings is relatively unknown. In the few studies that have followed participants after formal intervention, most people with MAFLD did not sustain their adherence to exercise and subsequently did not maintain the health benefits [117, 160, 161]. Effective strategies to promote long-term maintenance of exercise requires further investigation.

### Implementation of Recommendations into Practice

**Fig. 4:**
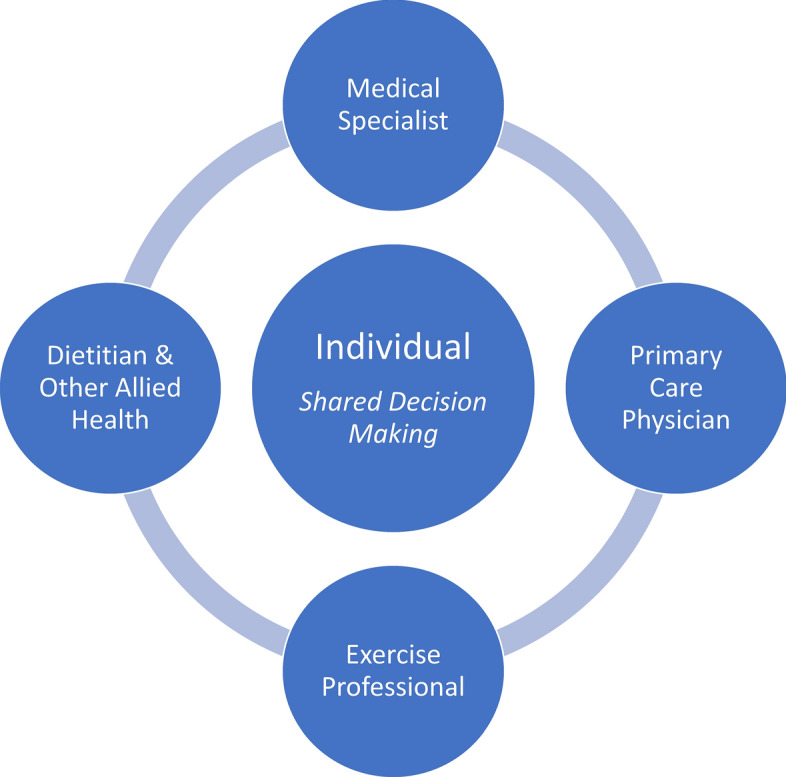
Patient-centred multi-disciplinary approach to MAFLD management

The broad management of MAFLD requires a patient-centred multi-disciplinary team approach (Fig. 4). Management approaches should encompass a biopsychosocial approach and emphasise the need to facilitate practical behavioural strategies that are individualised to the person’s local, cultural and socioeconomic circumstances, along with personal preferences and capabilities.

**Fig. 5:**
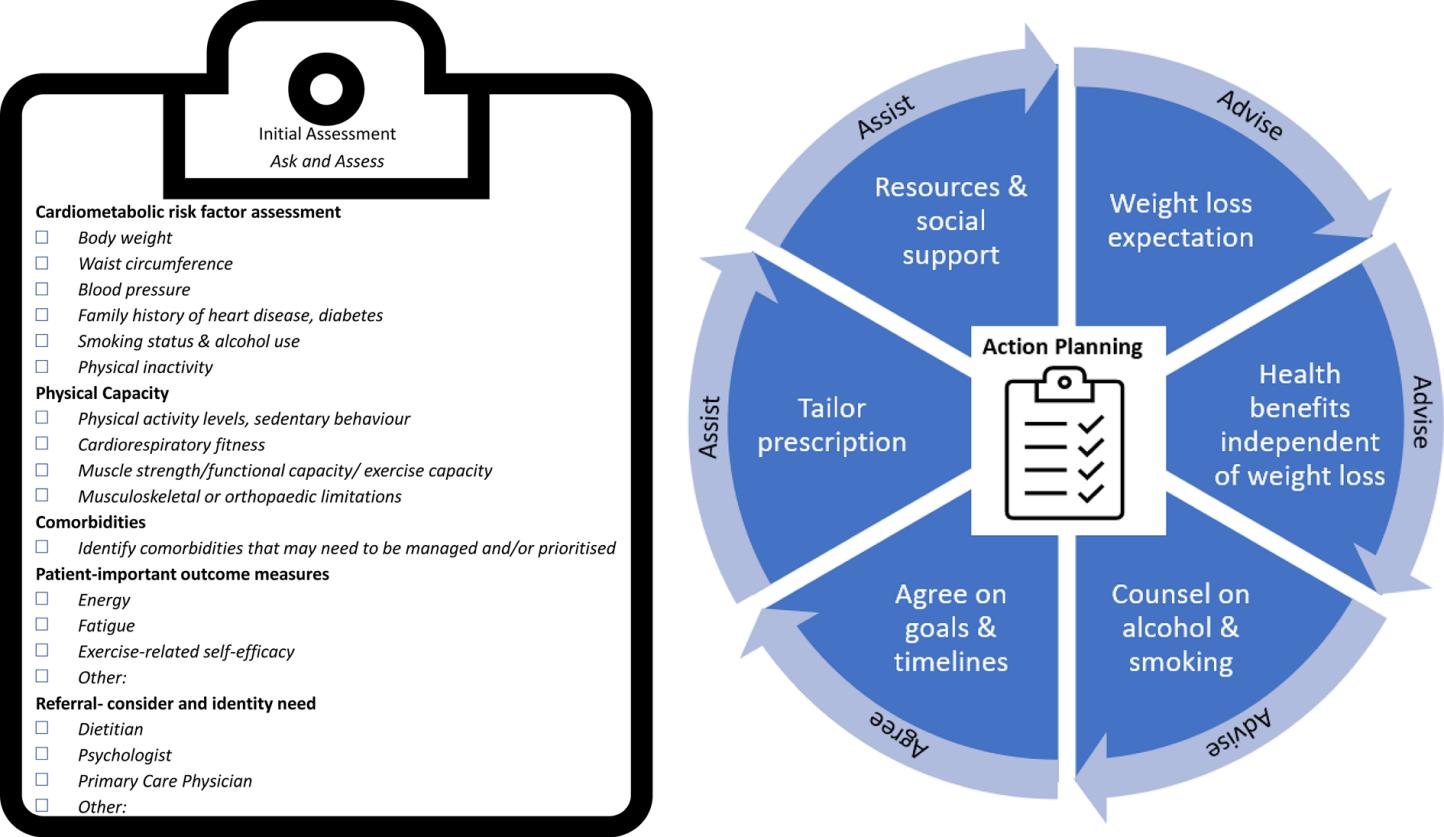
Framework for the implementation of exercise recommendations

A guideline implementation framework underpinned by the primary care ‘5As’ framework (ask and assess, advise, assist, arrange) is outlined in Fig. 5. For the exercise professional, initial assessments should encompass the suite of outcomes detailed in Sect. 1.4.2  that reflect the core goals of the broader team and can be pragmatically addressed within the context of exercise care.

Weight loss will be an important outcome for the broader multi-disciplinary management for most people with MAFLD. It is therefore critical to appropriately communicate the small weight loss typical with exercise intervention alone, particularly in the initial phase where exercise volume may be relatively low to accommodate low physical capacity. Failure to meet prescribed/expected weight loss targets has proved to be associated with programme drop out and feelings of guilt and failure [162], which may be detrimental to holistic treatment plans. As such, the multiple weight loss-independent benefits of exercise for people with MAFLD, and the central role of physical activity in weight-loss maintenance should be emphasised. For individuals in whom weight loss is a primary target, the practitioner should refer to specific recommendations [163] that provide guidance on the volume of exercise required for weight loss with exercise and consider referral a dietitian for concurrent dietary changes.

Asking about and advising on healthy lifestyle behaviours including alcohol use and smoking cessation should be included alongside other cardiometabolic disease risk factors, pertinent aspects of physical capacity and patient-identified outcomes of importance. If hazardous alcohol or smoking behaviour does not change sufficiently, or relapse occurs, referral back to the individual’s medical specialist or primary care physician for support is warranted.

Appropriate physical activity programming goals should be co-developed and agreed upon by the individual and practitioner. The recommendations in Table 2 should be used to develop both initial and target physical activity levels and exercise approaches with timelines for progression incorporated into the initial plan. These recommendations should be tailored to individual preferences and capabilities. Visual analogue scales or the goal attainment scale [42] may be useful to assess and document non-traditional clinical outcomes such as improvement in energy and fatigue, or other patient-identified outcomes of importance.

Resources to improve exercise-related self-efficacy (facilitated by scales such as the Self-Efficacy for Exercise Scale [39]) and exercise maintenance should be discussed and incorporated into programming. Behaviour change techniques that may support people with MAFLD include goal setting (e.g. via the Goal Attainment Scale [42]), self-monitoring of outcomes (e.g. waist circumference) and/or behaviours (e.g. via a pedometer, smartwatch-based application or other behaviour tracking application) and repeated contact with healthcare professionals. Support networks within an individual’s community, social and family group should be identified to facilitate accountability and social support.

### Barriers to Exercise Adoption and Adherence in MAFLD

A key barrier to exercise cited in the literature is a lack of information provision for patients highlighting the benefits of exercise in relation to treatment of MAFLD and a lack of personalised exercise advice/planning thereafter [164–168]. Patients have reported not knowing how to exercise/what to do, which resulted in a lack of confidence to commence [164, 169, 170]. Fatigue or lack of energy was commonly highlighted as a barrier to exercise for people with MAFLD [31, 164, 170–172], as was pain during exercise or the fear of exercise causing pain [31, 164, 172]. Fear of falling was also highlighted as a barrier [169], as were concerns that exercise would make other health conditions worse or that comorbidities would prevent them from exercising [167, 172]. Traditional barriers to exercise, such as lack of time [164, 167, 170, 172], cost [164, 172], access to equipment/facilities [164, 172], lack of support from family/friends to make changes [164, 165, 167] and work/family commitments [164, 167], were also frequently reported by people with MAFLD. A combination of these factors could lead to patients reporting a lack of willpower and/or motivation to exercise highlighted in some studies [167, 170]. This may be exacerbated by the high levels of anxiety and depression observed in this population [173], alongside the impact that MAFLD can have on emotional and cognitive functioning [31].

Exercise professionals have a key role in providing relevant information on the benefits of exercise in MAFLD and to alleviate individual patient anxieties about starting a tailored exercise programme. This may be particularly important in people with MAFLD given that it is a ‘silent’ condition with very few people experiencing symptoms, especially in earlier stages. Therefore, there may be reduced urgency to manage the condition and the exercise professional will have a role in education regarding the importance of managing MAFLD. Understanding the individual and societal barriers that people with MAFLD face in relation to exercise may help to enable personalised exercise interventions to be developed to maximise uptake and adherence.

## Special Considerations and Contraindications for Exercise in MAFLD

### Multiple Co-morbidities and Physical Limitations

People with MAFLD, and notably steatohepatitis, have lower health-related quality of life than the general population [174] and low engagement with physical activity [28, 31, 164, 175]. The pathophysiology underpinning MAFLD is also associated with exercise intolerance and diastolic and autonomic dysfunction [28, 176, 177], with the severity of impairment related to the severity of liver fibrosis [28]. Moreover, obesity and musculoskeletal issues are apparent in most people with MAFLD which may influence the selection of exercise modality. Comorbidities associated with MAFLD increase disease severity and the rate of disease progression to advanced liver disease or CVD. In the presence of one or more comorbidity (e.g. T2D, hypertension, chronic kidney disease) specific exercise guidelines pertaining to the comorbid condition(s) should be considered. Priority should be given to the condition(s) that represent the most immediate risk or present a barrier to achieving exercise targets. While this may mean that MAFLD-specific exercise targets need to be deferred or progressed towards, ideally MAFLD targets should be simultaneously met within this broader management approach. Exercise prescription should be individually tailored to incorporate both guidelines with modifications to exercise volume, intensity and modality as required to ensure safety.

### Medications

Although there are several promising agents for MAFLD in stage II and III clinical trials, at present there is no approved drug therapy [178]. However, while not specifically licenced for MAFLD, but relevant for the exercise professional to consider, medications that impact on glycaemic control and insulin sensitivity may be prescribed, particularly glucagon-like peptide 1 (GLP-1) agonists that improve glycaemic control and assist weight loss. While the first-line approach to MAFLD management centres on lifestyle intervention, it is likely that people with MAFLD will be prescribed medication as adjunctive therapy to manage cardiometabolic disease risk factors, and polypharmacy is common. Appropriate monitoring for hypoglycaemic or hypotensive events during or after exercise is necessary. Due to the interaction between exercise and some medications leading to additive effects, individuals being managed pharmacologically should be monitored for exercise-related adverse events and referred for medication review as required (see Sect. 1.4).

### Physical Activity/Exercise in People with Metabolic Steatohepatitis Related Cirrhosis

Liver cirrhosis (the late state of fibrosis/scarring of the liver) can be caused by several liver diseases and conditions. Cirrhosis can be classified as ‘compensated’ (e.g. the liver is coping with the damage and maintaining important functions; no overt symptoms) or ‘decompensated’ (liver function is compromised; symptoms of complications present).

Clinical features of hepatic decompensation stem from portal hypertension (found in late-stage cirrhosis), which in turn contributes to ascites, bleeding gastroesophageal varices (expanded blood vessels) and hepatic encephalopathy. All patients with cirrhosis (of any aetiology) are at increased risk of sarcopenia which can lead to frailty and loss of functional ability [179]. Maintaining functional status and improving quality of life are key treatment goals in this patient group.

Data from people with broad-aetiology cirrhosis indicate that exercise training is safe and effective for lowering portal pressures [180, 181], with further exercise prescription guidance published (see Tandon et al. [158] for prescription recommendations). However, there is a dearth of evidence for the efficacy of exercise specifically in people with metabolic steatohepatitis-related cirrhosis. Data from small studies of patients with early stage or well-compensated cirrhosis (mixed aetiologies) have shown that supervised and home exercise programmes, combining aerobic and resistance exercise, are safe, with no apparent increased risk of variceal haemorrhage or encephalopathy; these programmes also led to increases in lean body mass, reduction in overall adiposity, improved mobility and a marginal reduction in hepatic venous pressure gradient [182–185].

Evidence to inform exercise interventions for people with decompensated cirrhosis is lacking. Low-intensity activity is likely safe and feasible for those with decompensated cirrhosis, but it is important to liaise closely with other members of the multi-disciplinary team (including physician evaluation) before initiating an exercise programme for all patients with cirrhosis. This is to ensure complications of end-stage liver disease are being appropriately managed for safety reasons. For example, if the patient is at high risk of varices or has had a previous variceal bleed, relevant prophylaxis must be in place before exercise is prescribed. For further information on clinical considerations regarding these complications and recommendations for exercise assessment and prescription for people with cirrhosis (see Tandon et al. [158].

People with cirrhosis are hypercatabolic and may experience decreased appetite, early satiety and nutritional malabsorption. Nutritional assessment and optimisation should occur alongside the initiation of exercise to identify malnutrition, ensure that patients are meeting the appropriate protein and calorie requirements, and determine whether an increased energy intake is required due to becoming more physically active. Continued communication with the dietetic team should be maintained throughout [186].

## Conclusions

MAFLD is a highly prevalent chronic liver condition characterised by hepatic steatosis that is strongly linked with both cardiometabolic and liver-related sequalea. MAFLD management centres on lifestyle therapy (exercise and diet) driven by a patient-centred multi-disciplinary team. The broader management goals should target preventing liver disease progression and reversing MAFLD and reducing cardiovascular morbidity and mortality. The exercise professional plays a central care role in MAFLD management. There is strong evidence that aerobic exercise reduces hepatic steatosis with modest benefits (2–4%) and can be demonstrated irrespective of weight loss. At least 135 min/week and up to 240 min/week of moderate intensity aerobic activity is recommended for hepatic steatosis reduction, with no further benefit from completing higher intensity exercise (including HIIT approaches) so long as this volume of aerobic activity is achieved. This exercise dose is likely to result in broader health benefits. There is no clear evidence for a benefit of resistance training on outcomes for MAFLD beyond its established effects on lean mass maintenance, blood glucose control and neuromuscular strength. Resistance training should be considered in addition to, and not instead of, aerobic exercise targets. Exercise management should encompass shared decision making and engage practical behavioural strategies that are cognisant of an individual’s local, cultural and socioeconomic circumstances, along with personal preferences, comorbidities and physical capacity.

### Supplementary Information

Below is the link to the electronic supplementary material.

Supplementary file1 (DOCX 19 KB)

## Data Availability

Not applicable.
