# Supplementary material for: Exercise in the Management of Metabolic-Associated Fatty Liver Disease (MAFLD) in Adults: A Position Statement from Exercise and Sport Science Australia
Source: Sports Med. Author manuscript; Available in PMC 2024 Feb 7. (PMC10687186; doi:10.1007/s40279-023-01918-w)
Supplement: supplement [file NIHMS1932381-supplement-supplement.docx]

**Title:** Exercise in the management of metabolic-associated fatty liver disease (MAFLD) in adults: A position statement from Exercise and Sport Science Australia

**Authors:** Shelley E. Keating^1^, Angelo Sabag^2,3,4^, Kate Hallsworth^,5,6,7^, Ingrid J. Hickman^8,9^, Graeme A. Macdonald^9,10^, Jonathan G. Stine^11,12,13,14^, Jacob George^15^, Nathan A. Johnson^2,3^.

^1^ School of Human Movement and Nutrition Sciences, The University of Queensland, Brisbane, QLD, Australia

^2^Faculty of Medicine and Health, Discipline of Exercise and Sport Science, University of Sydney, Sydney, NSW, Australia

^3^Charles Perkins Centre, University of Sydney, Camperdown, NSW, Australia

^4^ NICM Health Research Institute, Western Sydney University, Westmead, NSW, Australia

^5^NIHR Newcastle Biomedical Research Centre, Newcastle Upon Tyne Hospitals NHS Foundation Trust, Newcastle Upon Tyne, UK

^6^Liver Unit, Newcastle Upon Tyne Hospitals NHS Foundation Trust, Newcastle Upon Tyne, UK

^7^Translational and Clinical Research Institute, Faculty of Medical Sciences, Newcastle University, Newcastle upon Tyne, UK

^8^Department of Nutrition and Dietetics, Princess Alexandra Hospital, Brisbane, QLD Australia.

^9^Faculty of Medicine, PA-Southside Clinical Unit, The University of Queensland, Brisbane, QLD Australia.

^10^Department of Gastroenterology and Hepatology, Princess Alexandra Hospital, Brisbane, QLD Australia

^11^Division of Gastroenterology and Hepatology, Department of Medicine, The Pennsylvania State University- Milton S. Hershey Medical Center, Hershey PA, USA

^12^Department of Public Health Sciences, The Pennsylvania State University- College of Medicine, Hershey PA, USA

^13^Liver Center, The Pennsylvania State University- Milton S. Hershey Medical Center, Hershey PA, USA

^14^Cancer Institute, The Pennsylvania State University- Milton S. Hershey Medical Center, Hershey, PA, USA

^15^Storr Liver Centre, The Westmead Institute for Medical Research and Westmead Hospital, University of Sydney, Sydney, NSW, Australia

**Corresponding author:**

Shelley E. Keating

Email: [s.keating@uq.edu.au](mailto:s.keating@uq.edu.au)

Address: Room 534, Bd 26B,

School of Human Movement and Nutrition Sciences

The University of Queensland, St Lucia, 4067

AUSTRALIA

ORCID: 0000-0001-5357-2721

**Online Resource 1:** Search Strategy

|  | Keywords |
| --- | --- |
| Exercise or sedentary behaviour | exercise OR HIIT OR high-intensity interval* OR high intensity interval* OR aerobic interval* OR HIT OR aerobic exercise OR endurance exercise OR aerobic training OR endurance training OR cardio training OR physical endurance OR physical exertion OR moderate intensity continuous training OR MICT OR sprint interval* OR strength training OR weight training OR resistance training OR progressive training OR progressive resistance OR weight lifting OR sedentary lifestyle OR sitting OR ((sedentary OR inactive) and (life* OR behave* OR lifestyle). |
| MAFLD | liver fat OR intrahepatic* OR non-alcoholic fatty liver disease OR NAFLD OR fatty liver OR hepatic steatosis OR hepatic OR liver OR steatohepatitis OR non-alcoholic steatohepatitis OR NASH OR aminotransferase OR AST OR ALT OR MAFLD OR metabolic-associated fatty liver disease OR metabolic-dysfunction associated fatty liver disease |
| Systematic Review | meta-analysis OR systematic review* OR meta-analys* |

| Database | Search |
| --- | --- |
| PubMed (combined final search) | ("meta-analysis"[Title/Abstract] OR "systematic review*"[Title/Abstract] OR "meta analys*"[Title/Abstract] OR "review*"[Title/Abstract]) AND ("Non-alcoholic Fatty Liver Disease"[MeSH Terms] OR ("liver fat"[Title/Abstract] OR "intrahepatic*"[Title/Abstract] OR "Non-alcoholic Fatty Liver Disease"[Title/Abstract] OR "NAFLD"[Title/Abstract] OR "fatty liver"[Title/Abstract] OR "hepatic steatosis"[Title/Abstract] OR "hepatic"[Title/Abstract] OR "liver"[Title/Abstract] OR "steatohepatitis"[Title/Abstract] OR "non alcoholic steatohepatitis"[Title/Abstract] OR "NASH"[Title/Abstract] OR "aminotransferase"[Title/Abstract] OR "AST"[Title/Abstract] OR "ALT"[Title/Abstract] OR "MAFLD"[Title/Abstract] OR "metabolic associated fatty liver disease"[Title/Abstract])) AND ("Exercise"[MeSH Terms] OR ("Exercise"[Title/Abstract] OR "HIIT"[Title/Abstract] OR "high intensity interval*"[Title/Abstract] OR "high intensity interval*"[Title/Abstract] OR "aerobic interval*"[Title/Abstract] OR "HIT"[Title/Abstract] OR "aerobic exercise"[Title/Abstract] OR "endurance exercise"[Title/Abstract] OR "aerobic training"[Title/Abstract] OR "endurance training"[Title/Abstract] OR "cardio training"[Title/Abstract] OR "physical endurance"[Title/Abstract] OR "physical exertion"[Title/Abstract] OR "moderate intensity continuous training"[Title/Abstract] OR "MICT"[Title/Abstract] OR "sprint interval*"[Title/Abstract] OR "strength training"[Title/Abstract] OR "weight training"[Title/Abstract] OR "resistance training"[Title/Abstract] OR "progressive training"[Title/Abstract] OR "progressive resistance"[Title/Abstract] OR "weight lifting"[Title/Abstract]) OR "sedentary"[Title/Abstract] OR "sitting"[Title/Abstract] OR (("sedentaries"[All Fields] OR "sedentariness"[All Fields] OR "sedentary"[All Fields] OR ("inactives"[All Fields] OR "sedentary behavior"[MeSH Terms] OR ("sedentary"[All Fields] AND "behavior"[All Fields]) OR "sedentary behavior"[All Fields] OR "inactive"[All Fields])) AND ("life*"[All Fields] OR "behave*"[All Fields] OR ("life style"[MeSH Terms] OR ("life"[All Fields] AND "style"[All Fields]) OR "life style"[All Fields] OR "lifestyle"[All Fields] OR "lifestyles"[All Fields])))) |
| Cochrane Library | (meta-analysis:ti,ab OR ("systematic" NEXT review*):ti,ab OR ("meta" NEXT analys*):ti,ab OR review*:ti,ab) AND ([mh "Non-alcoholic Fatty Liver Disease"] OR ("liver fat":ti,ab OR intrahepatic*:ti,ab OR "Non-alcoholic Fatty Liver Disease":ti,ab OR NAFLD:ti,ab OR "fatty liver":ti,ab OR "hepatic steatosis":ti,ab OR hepatic:ti,ab OR liver:ti,ab OR steatohepatitis:ti,ab OR "non alcoholic steatohepatitis":ti,ab OR NASH:ti,ab OR aminotransferase:ti,ab OR AST:ti,ab OR ALT:ti,ab OR MAFLD:ti,ab OR "metabolic associated fatty liver disease":ti,ab)) AND ([mh Exercise] OR (Exercise:ti,ab OR HIIT:ti,ab OR ("high intensity" NEXT interval*):ti,ab OR ("high intensity" NEXT interval*):ti,ab OR ("aerobic" NEXT interval*):ti,ab OR HIT:ti,ab OR "aerobic exercise":ti,ab OR "endurance exercise":ti,ab OR "aerobic training":ti,ab OR "endurance training":ti,ab OR "cardio training":ti,ab OR "physical endurance":ti,ab OR "physical exertion":ti,ab OR "moderate intensity continuous training":ti,ab OR MICT:ti,ab OR ("sprint" NEXT interval*):ti,ab OR "strength training":ti,ab OR "weight training":ti,ab OR "resistance training":ti,ab OR "progressive training":ti,ab OR "progressive resistance":ti,ab OR "weight lifting":ti,ab) OR sedentary:ti,ab OR sitting:ti,ab OR ((sedentaries OR sedentariness OR sedentary OR (inactives OR [mh "sedentary behavior"] OR (sedentary AND behavior) OR "sedentary behavior" OR inactive)) AND (life* OR behave* OR ([mh "life style"] OR (life AND style) OR "life style" OR lifestyle OR lifestyles)))) |
| Embase (Ovid) | (meta-analysis:ti,ab OR 'systematic review*':ti,ab OR 'meta analys*':ti,ab OR review*:ti,ab) AND ('Non-alcoholic Fatty Liver Disease'/exp OR ('liver fat':ti,ab OR intrahepatic*:ti,ab OR 'Non-alcoholic Fatty Liver Disease':ti,ab OR NAFLD:ti,ab OR 'fatty liver':ti,ab OR 'hepatic steatosis':ti,ab OR hepatic:ti,ab OR liver:ti,ab OR steatohepatitis:ti,ab OR 'non alcoholic steatohepatitis':ti,ab OR NASH:ti,ab OR aminotransferase:ti,ab OR AST:ti,ab OR ALT:ti,ab OR MAFLD:ti,ab OR 'metabolic associated fatty liver disease':ti,ab)) AND (Exercise/exp OR (Exercise:ti,ab OR HIIT:ti,ab OR 'high intensity interval*':ti,ab OR 'high intensity interval*':ti,ab OR 'aerobic interval*':ti,ab OR HIT:ti,ab OR 'aerobic exercise':ti,ab OR 'endurance exercise':ti,ab OR 'aerobic training':ti,ab OR 'endurance training':ti,ab OR 'cardio training':ti,ab OR 'physical endurance':ti,ab OR 'physical exertion':ti,ab OR 'moderate intensity continuous training':ti,ab OR MICT:ti,ab OR 'sprint interval*':ti,ab OR 'strength training':ti,ab OR 'weight training':ti,ab OR 'resistance training':ti,ab OR 'progressive training':ti,ab OR 'progressive resistance':ti,ab OR 'weight lifting':ti,ab) OR sedentary:ti,ab OR sitting:ti,ab OR ((sedentaries OR sedentariness OR sedentary OR (inactives OR 'sedentary behavior'/exp OR (sedentary AND behavior) OR 'sedentary behavior' OR inactive)) AND (life* OR behave* OR ('life style'/exp OR (life AND style) OR 'life style' OR lifestyle OR lifestyles)))) |
| CINAHL (EBSCO Host) | ((TI meta-analysis OR AB meta-analysis) OR (TI "systematic review*" OR AB "systematic review*") OR (TI "meta analys*" OR AB "meta analys*") OR (TI review* OR AB review*)) AND ((MH "Non-alcoholic Fatty Liver Disease+") OR ((TI "liver fat" OR AB "liver fat") OR (TI intrahepatic* OR AB intrahepatic*) OR (TI "Non-alcoholic Fatty Liver Disease" OR AB "Non-alcoholic Fatty Liver Disease") OR (TI NAFLD OR AB NAFLD) OR (TI "fatty liver" OR AB "fatty liver") OR (TI "hepatic steatosis" OR AB "hepatic steatosis") OR (TI hepatic OR AB hepatic) OR (TI liver OR AB liver) OR (TI steatohepatitis OR AB steatohepatitis) OR (TI "non alcoholic steatohepatitis" OR AB "non alcoholic steatohepatitis") OR (TI NASH OR AB NASH) OR (TI aminotransferase OR AB aminotransferase) OR (TI AST OR AB AST) OR (TI ALT OR AB ALT) OR (TI MAFLD OR AB MAFLD) OR (TI "metabolic associated fatty liver disease" OR AB "metabolic associated fatty liver disease"))) AND ((MH Exercise+) OR ((TI Exercise OR AB Exercise) OR (TI HIIT OR AB HIIT) OR (TI "high intensity interval*" OR AB "high intensity interval*") OR (TI "high intensity interval*" OR AB "high intensity interval*") OR (TI "aerobic interval*" OR AB "aerobic interval*") OR (TI HIT OR AB HIT) OR (TI "aerobic exercise" OR AB "aerobic exercise") OR (TI "endurance exercise" OR AB "endurance exercise") OR (TI "aerobic training" OR AB "aerobic training") OR (TI "endurance training" OR AB "endurance training") OR (TI "cardio training" OR AB "cardio training") OR (TI "physical endurance" OR AB "physical endurance") OR (TI "physical exertion" OR AB "physical exertion") OR (TI "moderate intensity continuous training" OR AB "moderate intensity continuous training") OR (TI MICT OR AB MICT) OR (TI "sprint interval*" OR AB "sprint interval*") OR (TI "strength training" OR AB "strength training") OR (TI "weight training" OR AB "weight training") OR (TI "resistance training" OR AB "resistance training") OR (TI "progressive training" OR AB "progressive training") OR (TI "progressive resistance" OR AB "progressive resistance") OR (TI "weight lifting" OR AB "weight lifting")) OR (TI sedentary OR AB sedentary) OR (TI sitting OR AB sitting) OR ((sedentaries OR sedentariness OR sedentary OR (inactives OR (MH "sedentary behavior+") OR (sedentary AND behavior) OR "sedentary behavior" OR inactive)) AND (life* OR behave* OR ((MH "life style+") OR (life AND style) OR "life style" OR lifestyle OR lifestyles)))) |
| Web of Science / multidisciplinary | (meta-analysis OR "systematic review*" OR "meta analys*" OR review*) AND ("Non-alcoholic Fatty Liver Disease" OR ("liver fat" OR intrahepatic* OR "Non-alcoholic Fatty Liver Disease" OR NAFLD OR "fatty liver" OR "hepatic steatosis" OR hepatic OR liver OR steatohepatitis OR "non alcoholic steatohepatitis" OR NASH OR aminotransferase OR AST OR ALT OR MAFLD OR "metabolic associated fatty liver disease")) AND (Exercise OR (Exercise OR HIIT OR "high intensity interval*" OR "high intensity interval*" OR "aerobic interval*" OR HIT OR "aerobic exercise" OR "endurance exercise" OR "aerobic training" OR "endurance training" OR "cardio training" OR "physical endurance" OR "physical exertion" OR "moderate intensity continuous training" OR MICT OR "sprint interval*" OR "strength training" OR "weight training" OR "resistance training" OR "progressive training" OR "progressive resistance" OR "weight lifting") OR sedentary OR sitting OR ((sedentaries OR sedentariness OR sedentary OR (inactives OR "sedentary behavior" OR (sedentary AND behavior) OR "sedentary behavior" OR inactive)) AND (life* OR behave* OR ("life style" OR (life AND style) OR "life style" OR lifestyle OR lifestyles)))) |
| SPORTDiscus (EBSCO Host) | ((TI "meta-analysis" OR AB "meta-analysis") OR (TI "systematic review*" OR AB "systematic review*") OR (TI "meta analys*" OR AB "meta analys*") OR (TI "review*" OR AB "review*")) AND (DE "Non-alcoholic Fatty Liver Disease" OR ((TI "liver fat" OR AB "liver fat") OR (TI "intrahepatic*" OR AB "intrahepatic*") OR (TI "Non-alcoholic Fatty Liver Disease" OR AB "Non-alcoholic Fatty Liver Disease") OR (TI "NAFLD" OR AB "NAFLD") OR (TI "fatty liver" OR AB "fatty liver") OR (TI "hepatic steatosis" OR AB "hepatic steatosis") OR (TI "hepatic" OR AB "hepatic") OR (TI "liver" OR AB "liver") OR (TI "steatohepatitis" OR AB "steatohepatitis") OR (TI "non alcoholic steatohepatitis" OR AB "non alcoholic steatohepatitis") OR (TI "NASH" OR AB "NASH") OR (TI "aminotransferase" OR AB "aminotransferase") OR (TI "AST" OR AB "AST") OR (TI "ALT" OR AB "ALT") OR (TI "MAFLD" OR AB "MAFLD") OR (TI "metabolic associated fatty liver disease" OR AB "metabolic associated fatty liver disease"))) AND (DE "Exercise" OR ((TI "Exercise" OR AB "Exercise") OR (TI "HIIT" OR AB "HIIT") OR (TI "high intensity interval*" OR AB "high intensity interval*") OR (TI "high intensity interval*" OR AB "high intensity interval*") OR (TI "aerobic interval*" OR AB "aerobic interval*") OR (TI "HIT" OR AB "HIT") OR (TI "aerobic exercise" OR AB "aerobic exercise") OR (TI "endurance exercise" OR AB "endurance exercise") OR (TI "aerobic training" OR AB "aerobic training") OR (TI "endurance training" OR AB "endurance training") OR (TI "cardio training" OR AB "cardio training") OR (TI "physical endurance" OR AB "physical endurance") OR (TI "physical exertion" OR AB "physical exertion") OR (TI "moderate intensity continuous training" OR AB "moderate intensity continuous training") OR (TI "MICT" OR AB "MICT") OR (TI "sprint interval*" OR AB "sprint interval*") OR (TI "strength training" OR AB "strength training") OR (TI "weight training" OR AB "weight training") OR (TI "resistance training" OR AB "resistance training") OR (TI "progressive training" OR AB "progressive training") OR (TI "progressive resistance" OR AB "progressive resistance") OR (TI "weight lifting" OR AB "weight lifting")) OR (TI "sedentary" OR AB "sedentary") OR (TI "sitting" OR AB "sitting") OR ((TX "sedentaries" OR TX "sedentariness" OR TX "sedentary" OR (TX "inactives" OR DE "sedentary behavior" OR (TX "sedentary" AND TX "behavior") OR TX "sedentary behavior" OR TX "inactive")) AND (TX "life*" OR TX "behave*" OR (DE "life style" OR (TX "life" AND TX "style") OR TX "life style" OR TX "lifestyle" OR TX "lifestyles")))) |
